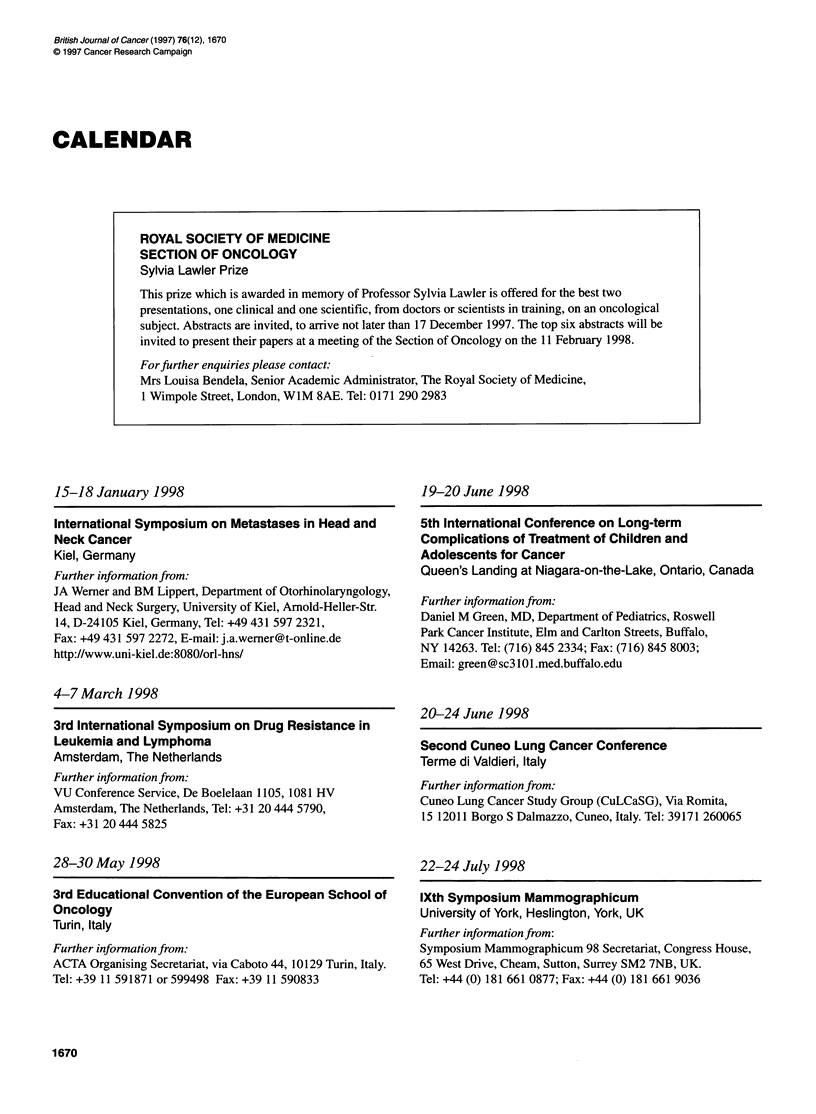# Calendar

**Published:** 1997

**Authors:** 


British Journal of Cancer (1997) 76(12), 1670
© 1997 Cancer Research Campaign

### CALENDAR

**ROYAL SOCIETY OF MEDICINE**
**SECTION OF ONCOLOGY**
Sylvia Lawler Prize

This prize which is awarded in memory of Professor Sylvia Lawler is offered for the best two presentations, one clinical and one scientific, from doctors or scientists in training, on an oncological subject. Abstracts are invited, to arrive not later than 17 December 1997. The top six abstracts will be invited to present their papers at a meeting of the Section of Oncology on the 11 February 1998.

*For further enquiries please contact:*
Mrs Louisa Bendela, Senior Academic Administrator, The Royal Society of Medicine, 1 Wimpole Street, London, W1M 8AE. Tel: 0171 290 2983

*15–18 January 1998*

**International Symposium on Metastases in Head and Neck Cancer**
Kiel, Germany

*Further information from:*
JA Werner and BM Lippert, Department of Otorhinolaryngology, Head and Neck Surgery, University of Kiel, Arnold-Heller-Str. 14, D-24105 Kiel, Germany, Tel: +49 431 597 2321, Fax: +49 431 597 2272, E-mail: j.a.werner@t-online.de http://www.uni-kiel.de:8080/orl-hns/

*4–7 March 1998*

**3rd International Symposium on Drug Resistance in Leukemia and Lymphoma**
Amsterdam, The Netherlands

*Further information from:*
VU Conference Service, De Boelelaan 1105, 1081 HV Amsterdam, The Netherlands, Tel: +31 20 444 5790, Fax: +31 20 444 5825

*28–30 May 1998*

**3rd Educational Convention of the European School of Oncology**
Turin, Italy

*Further information from:*
ACTA Organising Secretariat, via Caboto 44, 10129 Turin, Italy. Tel: +39 11 591871 or 599498 Fax: +39 11 590833

*19–20 June 1998*

**5th International Conference on Long-term Complications of Treatment of Children and Adolescents for Cancer**
Queen's Landing at Niagara-on-the-Lake, Ontario, Canada

*Further information from:*
Daniel M Green, MD, Department of Pediatrics, Roswell Park Cancer Institute, Elm and Carlton Streets, Buffalo, NY 14263. Tel: (716) 845 2334; Fax: (716) 845 8003; Email: green@sc3101.med.buffalo.edu

*20–24 June 1998*

**Second Cuneo Lung Cancer Conference**
Terme di Valdieri, Italy

*Further information from:*
Cuneo Lung Cancer Study Group (CuLCaSG), Via Romita, 15 12011 Borgo S Dalmazzo, Cuneo, Italy. Tel: 39171 260065

*22–24 July 1998*

**IXth Symposium Mammographicum**
University of York, Heslington, York, UK

*Further information from:*
Symposium Mammographicum 98 Secretariat, Congress House, 65 West Drive, Cheam, Sutton, Surrey SM2 7NB, UK. Tel: +44 (0) 181 661 0877; Fax: +44 (0) 181 661 9036